# Supplementary material for: Clinical Strains of Mycobacterium tuberculosis Representing Different Genotype Families Exhibit Distinct Propensities to Adopt the Differentially Culturable State
Source: Pathogens. 2024 Apr 12;13(4):318. doi: 10.3390/pathogens13040318 (PMC11054447; doi:10.3390/pathogens13040318)
Supplement: Supplementary file 1 [file pathogens-13-00318-s001.zip › Gordhan etal. Supplementary Table S1.pdf]

Table S1: Spoligotyping information used to classify the samples into the various *M. tuberculosis* genotype families and lineages.

Study	Participant Number	Binary code*	Family	Lineage	DCTB
BMG Study	59012	0000000000000000000000000000000000111111111<br>□□□□□□□□□□□□□□□□□□□□□□□□□□□□□□□□□□■■■■■■■■■	Beijing	Lineage 2 (East Asian)	Yes
	59014	0000000000000000000000000000000000111111111<br>□□□□□□□□□□□□□□□□□□□□□□□□□□□□□□□□□□■■■■■■■■■	Beijing		Yes
	59025	0000000000000000000000000000000000111111111<br>□□□□□□□□□□□□□□□□□□□□□□□□□□□□□□□□□□■■■■■■■■■	Beijing		No
	59032	0000000000000000000000000000000000111111111<br>□□□□□□□□□□□□□□□□□□□□□□□□□□□□□□□□□□■■■■■■■■■	Beijing		Yes
	59045	0000000000000000000000000000000000111111111<br>□□□□□□□□□□□□□□□□□□□□□□□□□□□□□□□□□□■■■■■■■■■	Beijing		Yes
	59046	0000000000000000000000000000000000111111111<br>□□□□□□□□□□□□□□□□□□□□□□□□□□□□□□□□□□■■■■■■■■■	Beijing		Yes
	59048	0000000000000000000000000000000000111111111<br>□□□□□□□□□□□□□□□□□□□□□□□□□□□□□□□□□□■■■■■■■■■	Beijing		Yes
	59050	0000000000000000000000000000000000111111111<br>□□□□□□□□□□□□□□□□□□□□□□□□□□□□□□□□□□■■■■■■■■■	Beijing		Yes
	59129	0000000000000000000000000000000000111111111<br>□□□□□□□□□□□□□□□□□□□□□□□□□□□□□□□□□□■■■■■■■■■	Beijing		Yes
	59130	0000000000000000000000000000000000111111111<br>□□□□□□□□□□□□□□□□□□□□□□□□□□□□□□□□□□■■■■■■■■■	Beijing		Yes
	57134	0000000000000000000000000000000000111111111<br>□□□□□□□□□□□□□□□□□□□□□□□□□□□□□□□□□□■■■■■■■■■	Beijing		Yes
	57135	0000000000000000000000000000000000111111111<br>□□□□□□□□□□□□□□□□□□□□□□□□□□□□□□□□□□■■■■■■■■■	Beijing		No
	57146	0000000000000000000000000000000000111111111<br>□□□□□□□□□□□□□□□□□□□□□□□□□□□□□□□□□□■■■■■■■■■	Beijing		Yes
	57154	0000000000000000000000000000000000111111111<br>□□□□□□□□□□□□□□□□□□□□□□□□□□□□□□□□□□■■■■■■■■■	Beijing		Yes
	57165	0000000000000000000000000000000000111111111<br>□□□□□□□□□□□□□□□□□□□□□□□□□□□□□□□□□□■■■■■■■■■	Beijing		Yes
MGIT-Plus Study	MP060	0000000000000000000000000000000000111111111<br>□□□□□□□□□□□□□□□□□□□□□□□□□□□□□□□□□□■■■■■■■■■	Beijing		Yes
	MP063	0000000000000000000000000000000000111111111<br>□□□□□□□□□□□□□□□□□□□□□□□□□□□□□□□□□□■■■■■■■■■	Beijing		Yes
	MP064	0000000000000000000000000000000000111111111<br>□□□□□□□□□□□□□□□□□□□□□□□□□□□□□□□□□□■■■■■■■■■	Beijing		No
	MP078	0000000000000000000000000000000000111111111<br>□□□□□□□□□□□□□□□□□□□□□□□□□□□□□□□□□□■■■■■■■■■	Beijing		Yes
	MP081	0000000000000000000000000000000000111111111<br>□□□□□□□□□□□□□□□□□□□□□□□□□□□□□□□□□□■■■■■■■■■	Beijing		Yes
	MP090	0000000000000000000000000000000000111111111<br>□□□□□□□□□□□□□□□□□□□□□□□□□□□□□□□□□□■■■■■■■■■	Beijing		Yes
	MP091	0000000000000000000000000000000000111111111<br>□□□□□□□□□□□□□□□□□□□□□□□□□□□□□□□□□□■■■■■■■■■	Beijing		Yes
	MP093	0000000000000000000000000000000000111111111<br>□□□□□□□□□□□□□□□□□□□□□□□□□□□□□□□□□□■■■■■■■■■	Beijing		No
	MP095	0000000000000000000000000000000000111111111<br>□□□□□□□□□□□□□□□□□□□□□□□□□□□□□□□□□□■■■■■■■■■	Beijing		Yes
	MP096	0000000000000000000000000000000000111111111<br>□□□□□□□□□□□□□□□□□□□□□□□□□□□□□□□□□□■■■■■■■■■	Beijing		Yes
	MP101	0000000000000000000000000000000000111111111<br>□□□□□□□□□□□□□□□□□□□□□□□□□□□□□□□□□□■■■■■■■■■	Beijing		Yes
	MP103	0000000000000000000000000000000000111111111<br>□□□□□□□□□□□□□□□□□□□□□□□□□□□□□□□□□□■■■■■■■■■	Beijing		Yes

BMG Study	59003	1111111100111001111000111110111000001101100<br>■■■■■■■■□□■■■□□■■■■□□□■■■■■□■■■□□□□□■■□■■□□	T	Lineage 4 (Euro American)	Yes
	59005	1111111111111111111111111111111100001111111<br>■■■■■■■■■■■■■■■■■■■■■■■■■■■■■■■■□□□□■■■■■■■	T		Yes
	59015	1111111110111111111100101101111000001010111<br>■■■■■■■■■□■■■■■■■■■■□□■□■■□■■■■□□□□□■□■□■■■	T1		Yes
	59017	1111111101111111110011111011111000001001011<br>■■■■■■■■□■■■■■■■■■□□■■■■■□■■■■■□□□□□■□□■□■■	T		No
	59018	1111111111111111111100111111111100001111111<br>■■■■■■■■■■■■■■■■■■■■□□■■■■■■■■■■□□□□■■■■■■■	T		No
	59019	1111111111101100000000001110111100001110110<br>■■■■■■■■■■■□■■□□□□□□□□□□■■■□■■■■□□□□■■■□■■□	T5		Yes
	59021	1111111111101111111111001110111100001110110<br>■■■■■■■■■■■□■■■■■■■■■■□□■■■□■■■■□□□□■■■□■■□	T2		Yes
	59029	1111111111111111111100111110001100001101111<br>■■■■■■■■■■■■■■■■■■■■□□■■■■■□□□■■□□□□■■□■■■■	T		No
	59034	1111111111111111111101111110111100001111111<br>■■■■■■■■■■■■■■■■■■■■□■■■■■■□■■■■□□□□■■■■■■■	T		No
	59055	1011111111111111111001111111111100001111111<br>■□■■■■■■■■■■■■■■■■■□□■■■■■■■■■■■□□□□■■■■■■■	T		Yes
	59056	1011111111111111111001111111111100001111111<br>■□■■■■■■■■■■■■■■■■■□□■■■■■■■■■■■□□□□■■■■■■■	T		Yes
	59057	1011111111111111111001111111111100001111111<br>■□■■■■■■■■■■■■■■■■■□□■■■■■■■■■■■□□□□■■■■■■■	T		No
	59127	1011111111111111111101111111111100001111111<br>■□■■■■■■■■■■■■■■■■■■□■■■■■■■■■■■□□□□■■■■■■■	T		Yes
	59128	1011111111111111111001111111111100001111111<br>■□■■■■■■■■■■■■■■■■■□□■■■■■■■■■■■□□□□■■■■■■■	T		Yes
	57158	1111111111111111111001111111111100001111111<br>■■■■■■■■■■■■■■■■■■■□□■■■■■■■■■■■□□□□■■■■■■■	T		Yes
	57162	1111111111111111111111111110111100001111110<br>■■■■■■■■■■■■■■■■■■■■■■■■■■■□■■■■□□□□■■■■■■□	T		Yes
MGIT-Plus Study	MP042	1111111111111111111111111111110100001110011<br>■■■■■■■■■■■■■■■■■■■■■■■■■■■■■■□■□□□□■■■□□■■	T2		No
	MP043	1111111111111111111111111110111100001111110<br>■■■■■■■■■■■■■■■■■■■■■■■■■■■□■■■■□□□□■■■■■■□	T		No
	MP045	1111111111111111111111111110111100001111110<br>■■■■■■■■■■■■■■■■■■■■■■■■■■■□■■■■□□□□■■■■■■□	T		No
	MP052	0000000000000000000001111111111100001111111<br>□□□□□□□□□□□□□□□□□□□□□■■■■■■■■■■■□□□□■■■■■■■	T1		No
	MP061	1111111101111111111000011111111100001100111<br>■■■■■■■■□■■■■■■■■■■□□□□■■■■■■■■■□□□□■■□□■■■	T		No
	MP073	1110011111111111111111111111111100001111111<br>■■■□□■■■■■■■■■■■■■■■■■■■■■■■■■■■□□□□■■■■■■■	T		No
	MP088	1111111111111111111101111111111100001111111<br>■■■■■■■■■■■■■■■■■■■■□■■■■■■■■■■■□□□□■■■■■■■	T		No
	MP089	1111111111111111111101111111111100001111111<br>■■■■■■■■■■■■■■■■■■■■□■■■■■■■■■■■□□□□■■■■■■■	T		Yes
	MP092	1111111111111111111001111111111100001111111<br>■■■■■■■■■■■■■■■■■■■□□■■■■■■■■■■■□□□□■■■■■■■	T		Yes
	MP097	1111111111111111111101111111111100001111111<br>■■■■■■■■■■■■■■■■■■■■□■■■■■■■■■■■□□□□■■■■■■■	T		No
	MP098	1111111111111111111111111111111100001111110<br>■■■■■■■■■■■■■■■■■■■■■■■■■■■■■■■■□□□□■■■■■■□	T		Yes
	MP100	1111111111111111111111111111111100001111111<br>■■■■■■■■■■■■■■■■■■■■■■■■■■■■■■■■□□□□■■■■■■■	T		Yes
	MP102	1111111111111111111111111111111100001111111<br>■■■■■■■■■■■■■■■■■■■■■■■■■■■■■■■■□□□□■■■■■■■	T		Yes
	MP104	1111111111111111111111111111111100001111111<br>■■■■■■■■■■■■■■■■■■■■■■■■■■■■■■■■□□□□■■■■■■■	T		Yes
	MP106	1111111111111111111111111111111100001111111	T1		No

		■■■■■■■■■■■■■■■■■■■■■■■■■■■■■■■■□□□□■■■■■■■			
	MP107	1111111101111111110011111011111000001001011<br>■■■■■■■■□■■■■■■■■■□□■■■■■□■■■■■□□□□□■□□■□■■	T		No
	MP112	1111111111111111111111111111111100001111111<br>■■■■■■■■■■■■■■■■■■■■■■■■■■■■■■■■□□□□■■■■■■■	T1		No
	MP115	1111111111111111111111111111111100001111111<br>■■■■■■■■■■■■■■■■■■■■■■■■■■■■■■■■□□□□■■■■■■■	T1		No
	MP118	1111111111111111111111111111111100001111111<br>■■■■■■■■■■■■■■■■■■■■■■■■■■■■■■■■□□□□■■■■■■■	T1		No
	MP119	1111111111111111111111111111111100001111111<br>■■■■■■■■■■■■■■■■■■■■■■■■■■■■■■■■□□□□■■■■■■■	T1		No
**BMG Study**	59131	1111111100111111111111111111111100001111110<br>■■■■■■■■□□■■■■■■■■■■■■■■■■■■■■■■□□□□■■■■■■□	S-family	**Lineage 4 (Euro American)**	Yes
	57139	1011111100111111111111111111111100001111111<br>■□■■■■■■□□■■■■■■■■■■■■■■■■■■■■■■□□□□■■■■■■□	S-family		No
	57140	1111111100111111111111111110111100001111110<br>■■■■■■■■□□■■■■■■■■■■■■■■■■■□■■■■□□□□■■■■■■□	S-family		Yes
	57142	1011111100111111111111111111111100001111111<br>■□■■■■■■□□■■■■■■■■■■■■■■■■■■■■■■□□□□■■■■■■□	S-family		Yes
	57144	1111111100111111111111111111111110000111111<br>■■■■■■■■□□■■■■■■■■■■■■■■■■■■■■■■□□□□■■■■■■■	S-family		Yes
	57148	1111111100111111111111111111111110000111111<br>■■■■■■■■□□■■■■■■■■■■■■■■■■■■■■■■□□□□■■■■■■■	S-family		No
	57151	1111111100111111111111111111111110000111111<br>■■■■■■■■□□■■■■■■■■■■■■■■■■■■■■■■□□□□■■■■■■■	S-family		No
	57159	1111111100111111111111111111111110000111111<br>■■■■■■■■□□■■■■■■■■■■■■■■■■■■■■■■□□□□■■■■■■■	S-family		Yes
	57160	1111111100111111111111111110111100001111110<br>■■■■■■■■□□■■■■■■■■■■■■■■■■■□■■■■□□□□■■■■■■□	S-family		Yes
	57161	1111111100111111111111111110111100001111110<br>■■■■■■■■□□■■■■■■■■■■■■■■■■■□■■■■□□□□■■■■■■□	S-family		Yes
	57163	1111111100111111111111111111111100001111110<br>■■■■■■■■□□■■■■■■■■■■■■■■■■■□■■■■□□□□■■■■■■□	S-family		Yes
	57170	1111111000011111111111111111100000000111111<br>■■■■■■■□□□□■■■■■■■■■■■■■■■■■■□□□□□□□□■■■■■■	S-family		No
	57172	1111111000011111111111111111100000000111111<br>■■■■■■■□□□□■■■■■■■■■■■■■■■■■■□□□□□□□□■■■■■■	S-family		Yes
	57173	1111111000011111111111111111100000000111111<br>■■■■■■■□□□□■■■■■■■■■■■■■■■■■■□□□□□□□□■■■■■■	S-family		Yes
**MGIT-Plus Study**	MP039	1111111100111111111111111111111100001111111<br>■■■■■■■■□□■■■■■■■■■■■■■■■■■■■■■■□□□□■■■■■■■	S-family		No
	MP048	1111111100111111111111111111111100001111111<br>■■■■■■■■□□■■■■■■■■■■■■■■■■■■■■■■□□□□■■■■■■■	S-family		No
	MP049	1111111100111111111111111111111100001111111<br>■■■■■■■■□□■■■■■■■■■■■■■■■■■■■■■■□□□□■■■■■■■	S-family		No
	MP070	1111111100111111111111111111111100001111111<br>■■■■■■■■□□■■■■■■■■■■■■■■■■■■■■■■□□□□■■■■■■■	S-family		No
**BMG Study**	59007	1111111100000011111110000001111110000111110<br>■■■■■■■■□□□□□□■■■■■■■□□□□□□■■■■■■□□□□■■■■■□	LAM12	**Lineage 4 (Euro American)**	Yes
	59016	1111111000001111111100000111011110000111110<br>■■■■■■■□□□□□■■■■■■■■□□□□□■■■□■■■■□□□□■■■■■□	LAM3		No
	59004	1111111111111111111100001100001100001111111<br>■■■■■■■■■■■■■■■■■■■■□□□□■■□□□□■■□□□□■■■■■■■	LAM11		Yes
	59002	1111111111100000111000001011111100001001111<br>■■■■■■■■■■■□□□□□■■■□□□□□■□■■■■■■□□□□■□□■■■■	LAM5		Yes
	59024	1011111111100000111000001011111100001001111<br>■□■■■■■■■■■□□□□□■■■□□□□□■□■■■■■■□□□□■□□■■■■	LAM5		Yes
	59043	1111111111111111111100001100001100001100111<br>■■■■■■■■■■■■■■■■■■■■□□□□■■□□□□■■□□□□■■□□■■■	LAM11		Yes
	59028	1011111111100000111000001011111100001001111<br>■□■■■■■■■■■□□□□□■■■□□□□□■□■■■■■■□□□□■□□■■■■	LAM5		Yes
	59044	1011111111111111111100001111111100001110111	LAM4		No

		■□■■■■■■■■■■■■■■■■■■■□□□□■■■■■■■■■□□□□■■■□■■■			
	57149	11111111111111111001100001100001100001110110<br>■■■■■■■■■■■■■■■■■□□■■□□□□■■□□□□■■□□□□■■■□■■□	LAM11		Yes
	57150	10111111111100000111000001011111100001001111<br>■□■■■■■■■■■■■□□□□□■■■□□□□□■□■■■■■■□□□□■□□■■■■	LAM5		Yes
**MGIT-Plus Study**	MP044	11111111111111111111100001111111100001111111<br>■■■■■■■■■■■■■■■■■■■■■□□□□■■■■■■■■□□□□■■■■■■■	LAM		No
	MP053	11111111000001111111000011111111100001111111<br>■■■■■■■■□□□□□■■■■■■■□□□□■■■■■■■■■□□□□■■■■■■■	LAM3		No
	MP054	11111111000001111111000011111111100001111111<br>■■■■■■■■□□□□□■■■■■■■□□□□■■■■■■■■■□□□□■■■■■■■	LAM3		No
	MP055	11001111000001111111000011111111100001111111<br>■■□□■■■■□□□□□■■■■■■■□□□□■■■■■■■■■□□□□■■■■■■■	LAM3		No
	MP058	11111111000001111111000011111111100001111111<br>■■■■■■■■□□□□□■■■■■■■□□□□■■■■■■■■■□□□□■■■■■■■	LAM3		No
	MP079	11111100101110011111000011111111100001101111<br>■■■■■■□□■□■■■□□■■■■■□□□□■■■■■■■■■□□□□■■□■■■■	LAM		No
	MP094	11111100101110011111000011111111100001101111<br>■■■■■■□□■□■■■□□■■■■■□□□□■■■■■■■■■□□□□■■□■■■■	LAM		No
	MP105	11111100101110011111000011111111100001101111<br>■■■■■■□□■□■■■□□■■■■■□□□□■■■■■■■■■□□□□■■□■■■■	LAM		Yes
**BMG Study**	59010	11100000000001111101111111110111100001111110<br>■■■□□□□□□□□□□■■■■■□■■■■■■■■■□■■■■□□□□■■■■■■□	X3	**Lineage 4 (Euro American)**	No
	59030	10100000000000111011111111111111100001111111<br>■□■□□□□□□□□□□□□□■■■□■■■■■■■■■■■■■■■□□□□■■■■■■■	X3		Yes
	59031	11100000000001111101111111000111100001111111<br>■■■□□□□□□□□□□■■■■■□■■■■■■■□□□■■■■□□□□■■■■■■■	X3		Yes
	59035	10100000000001111101111111111111100001111111<br>■□■□□□□□□□□□□■■■■■□■■■■■■■■■■■■■■■□□□□■■■■■■■	X3		Yes
	59052	11100000000001111101111111000111100001111111<br>■■■□□□□□□□□□□■■■■■□■■■■■■■□□□■■■■□□□□■■■■■■■	X3		Yes
	57152	11111111111111111011111111101111100001111110<br>■■■■■■■■■■■■■■■■■□■■■■■■■■■□■■■■□□□□■■■■■■□	X1		No
**MGIT-Plus Study**	MP068	11100000000001111101111111111111100001111111<br>■■■□□□□□□□□□□■■■■■□■■■■■■■■■■■■■■■□□□□■■■■■■■	X3		No
	MP069	11100000000001111101111111111111100001111111<br>■■■□□□□□□□□□□■■■■■□■■■■■■■■■■■■■■■□□□□■■■■■■■	X3		Yes
	MP077	11100000000000111011111111111111100001111111<br>■■■□□□□□□□□□□□□■■■□■■■■■■■■■■■■■■■□□□□■■■■■■■	X3		Yes
	MP110	11100000000001111101111111111111100001111111<br>■■■□□□□□□□□□□■■■■■□■■■■■■■■■■■■■■■□□□□■■■■■■■	X3		Yes

*Inna Vitol, Jeffrey Driscoll, Barry Kreiswirth, Natalia Kurepina, Kristin P. Bennett, "Identifying *Mycobacterium tuberculosis* Complex Strain Families using Spoligotypes", Infection, Genetics and Evolution, Volume 6, Issue 6, November 2006, Pages 491-504." in publications that benefit from this tool. https://tbinsight.cs.rpi.edu/run_tb_ lineage.html
