# Supplementary figures and images for: Clinical Strains of Mycobacterium tuberculosis Representing Different Genotype Families Exhibit Distinct Propensities to Adopt the Differentially Culturable State
Source: Pathogens. 2024 Apr 12;13(4):318. doi: 10.3390/pathogens13040318 (PMC11054447; doi:10.3390/pathogens13040318)

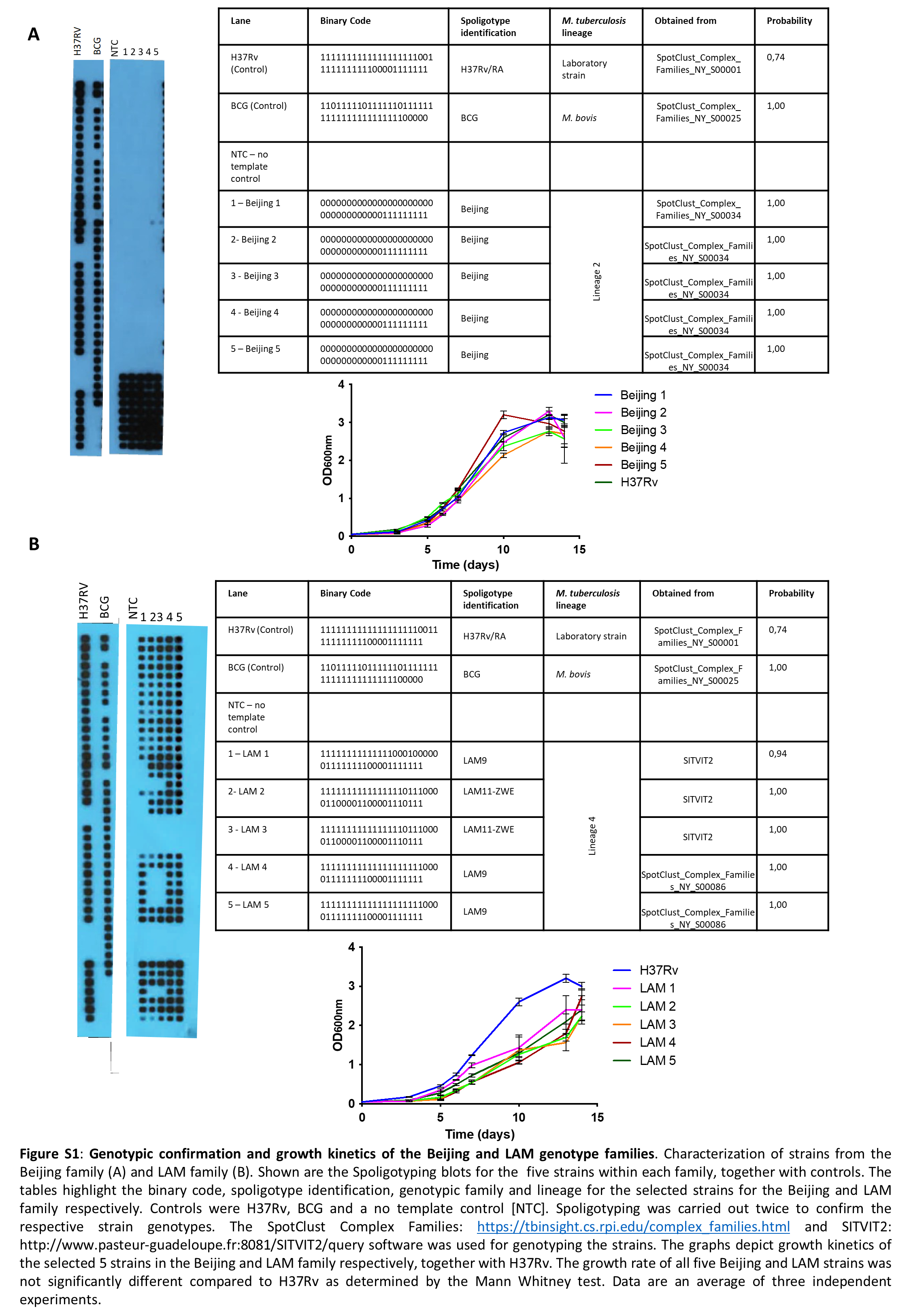

Supplement: Supplementary file 1 [file pathogens-13-00318-s001.zip › Figure S1.TIF]

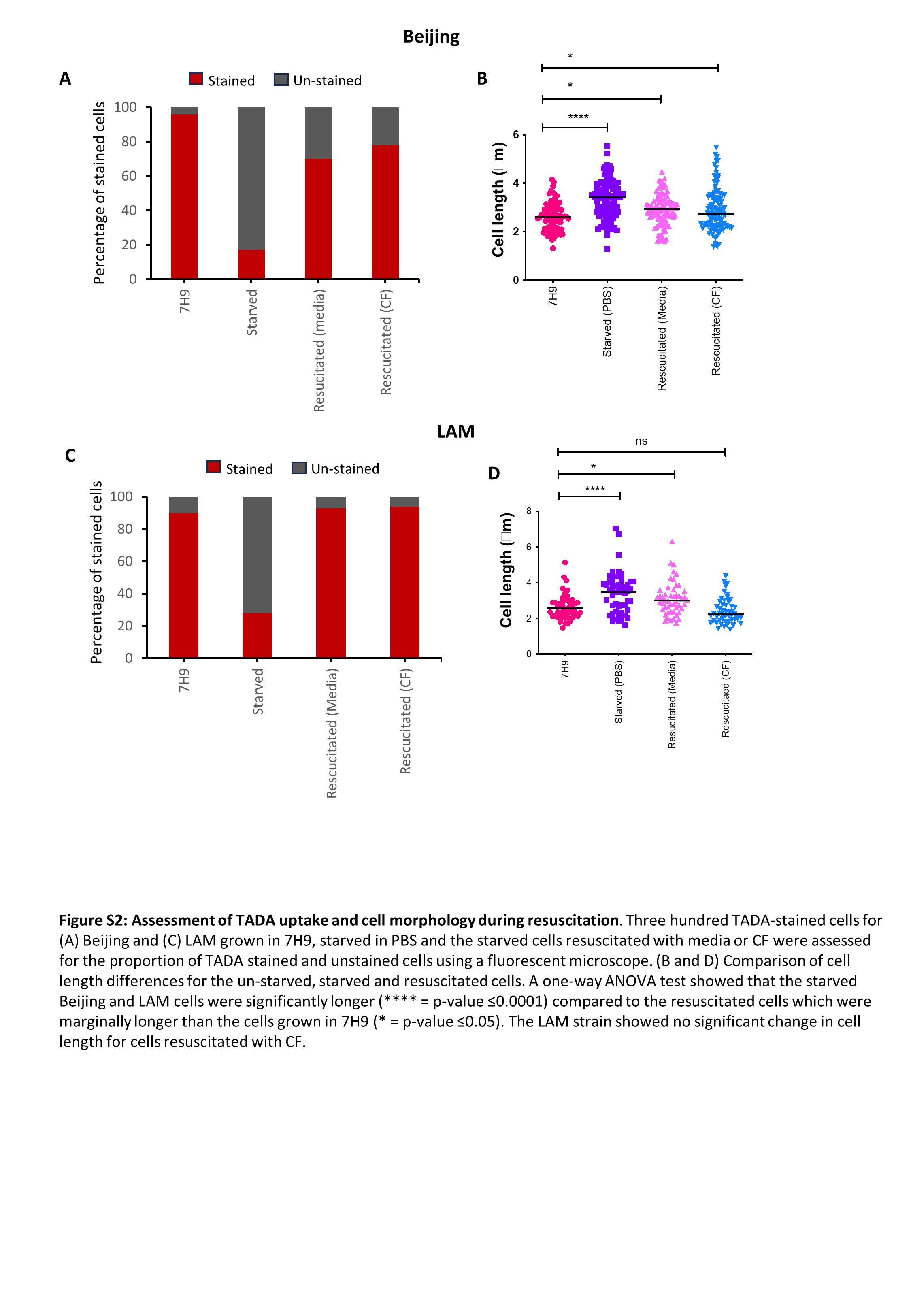

Supplement: Supplementary file 1 [file pathogens-13-00318-s001.zip › Figure S2.TIF]

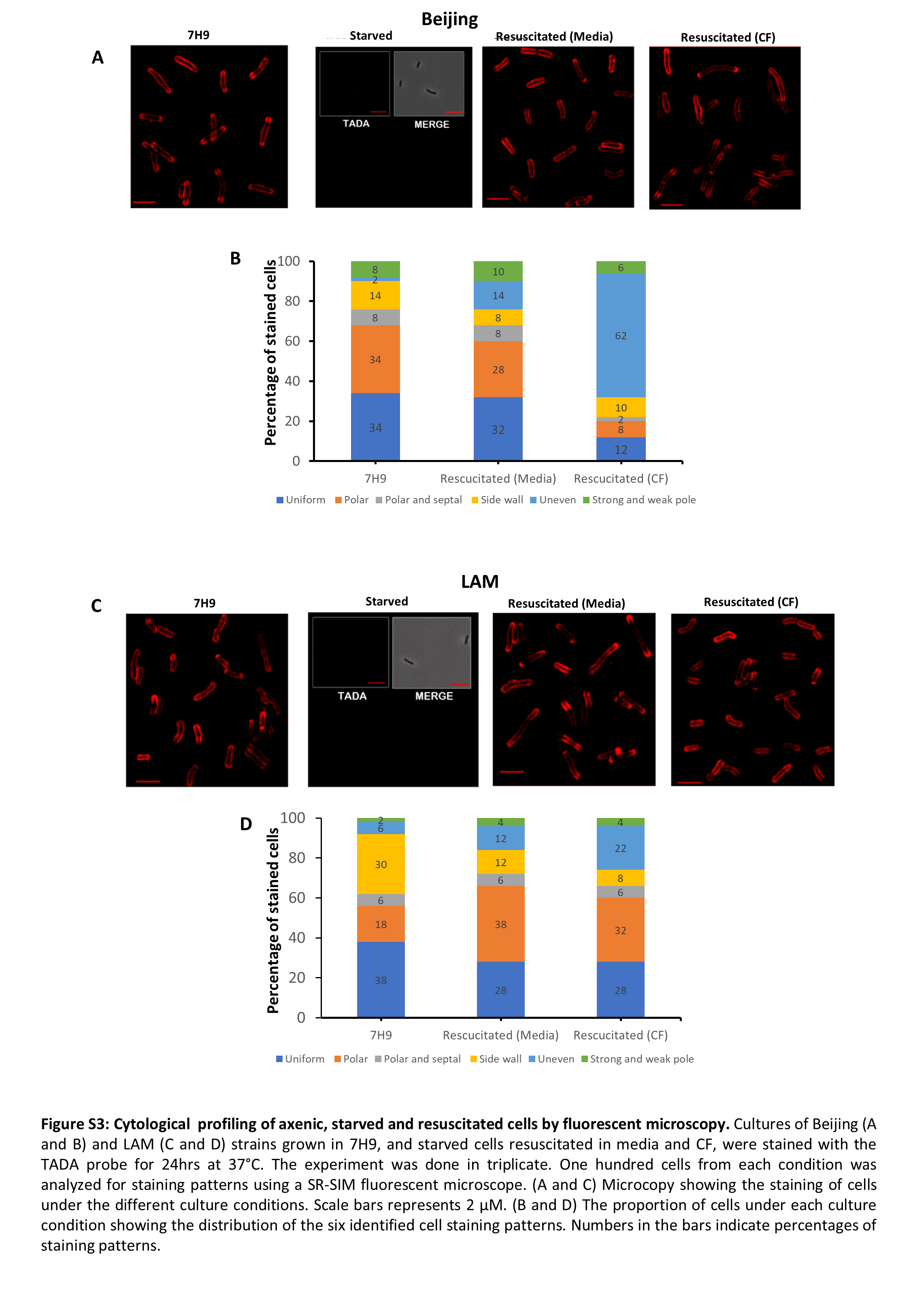

Supplement: Supplementary file 1 [file pathogens-13-00318-s001.zip › Figure S3.TIF]
